# Knowledge into the Practice against COVID-19: A Cross-Sectional Study from Ghana

**DOI:** 10.3390/ijerph182412902

**Published:** 2021-12-07

**Authors:** Prince Yeboah, Dennis Bomansang Daliri, Ahmad Yaman Abdin, Emmanuel Appiah-Brempong, Werner Pitsch, Anto Berko Panyin, Emmanuel Bentil Asare Adusei, Afraa Razouk, Muhammad Jawad Nasim, Claus Jacob

**Affiliations:** 1 Division of Bioorganic Chemistry, School of Pharmacy, Saarland University, 66123 Saarbruecken, Germany; s8pryebo@stud.uni-saarland.de (P.Y.); yaman.abdin@uni-saarland.de (A.Y.A.); afra00001@stud.uni-saarland.de (A.R.); jawad.nasim@uni-saarland.de (M.J.N.); 2 Department of Psychiatry, Komfo Anokye Teaching Hospital, Kumasi, Ghana; dennisuds@gmail.com; 3 Department of Health Promotion & Education, School of Public Health, Kwame Nkrumah University of Science & Technology, Kumasi, Ghana; ebrempong.chs@knust.edu.gh; 4 Department for Economics and Sociology of Sports, Faculty of Economics and Empirical Human Sciences, Institute of Sport Sciences, Saarland University, 66123 Saarbruecken, Germany; we.pitsch@mx.uni-saarland.de; 5 Department of Pharmacy Practice, Faculty of Pharmacy and Pharmaceutical Sciences, Kwame Nkrumah University of Science & Technology, Kumasi, Ghana; 6 Department of Pharmaceutical Chemistry, Faculty of Pharmacy & Pharmaceutical Sciences, Kwame Nkrumah University of Science & Technology, Kumasi, Ghana; bentil.emmanuelasare@gmail.com

**Keywords:** attitudes, COVID-19, Ghana, knowledge of COVID-19, linear multiple regression analysis, practices against COVID-19, preventive measures, public health, socio-demographics, vaccination

## Abstract

The COVID-19 pandemic has affected populations globally, including Ghana. Knowledge of the COVID-19 disease, and the application of preventive public health interventions are pivotal to its control. Besides a lockdown, measures taken against the spread of the virus include the wearing of face masks, social distancing, regular hand washing with soap and, more recently, vaccination against the virus. In order to establish a possible link between the knowledge of the disease and compliance with preventive measures, including vaccination, a cross-sectional study employing an interview-structured questionnaire was conducted in six regions of Ghana (*n* = 1560). An adequate level of knowledge of COVID-19 (69.9%) was reported. The linear multiple regression analysis further explicated the differences in the knowledge of COVID-19 among the respondents by their knowledge of cholera and influenza (adjusted R-Square = 0.643). Despite this profound knowledge of the illness, two thirds of the respondents were unwilling to follow basic preventive measures and only 35.3% were willing to be vaccinated. Amazingly, neither knowledge of COVID-19 nor the socio-demographic characteristics had any meaningful influence on the practice of preventive measures. Personal attitude leading to efficient public compliance with preventive measures, therefore, is a critical issue demanding special attention and effective interventions by the government and locals with authority to curb the spread of the pandemic which surpasses the traditional channels of public health communication. This includes a roll-out of persuasion, possibly including public figures and influencers, and in any case, a balanced and open discussion addressing the acceptance of the COVID-19 vaccine in order to avoid new variants and comparable problems currently facing many countries of Western Europe. Indeed, a profound hesitancy against vaccination may turn African countries such as Ghana for many years into hotspots of new viral variants.

## 1. Introduction

Since its first appearance in December 2019, COVID-19 has become a global pandemic and the virus is also increasingly affecting countries in Africa, which have witnessed viral and bacterial outbreaks, such as H1N1 influenza and cholera, before. On February 2020, researchers projected that developing countries, such as the ones in sub-Saharan Western and Central Africa, may be unable to mount an adequate response in terms of infection control and disease management, owing to a poor healthcare structure and lack of resources [1,2]. Although the outbreak of Ebola in countries, such as Nigeria, Liberia and the Democratic Republic of Congo in 2014–2016, may have highlighted the necessity to prepare the regional defenses against such pandemics, few concrete measures had been taken since. Indeed, the recent COVID-19 outbreak indicates that these limited efforts have been confronted off-guard and put to the test [3].

Nonetheless, the impact of COVID-19 in Africa has been country specific and often surprisingly contained [4,5]. While countries such as South Africa have suffered an outbreak eventually leading to new variants of SARS-CoV-2, in sub-Saharan African countries such as Ghana, with a population of 30 million, the impact of the virus has been so far relatively modest [6]. Ghana recorded her first two cases of COVID-19 in March of 2020, comparatively late in the early days of the pandemic. Since March 2020, there has been a gradual and occasional sharp increase in the number of confirmed cases. As of November 2021, Ghana has recorded a total of 131,000 confirmed cases of COVID-19 and 1208 fatalities, while Poland, a European country with a similar population, suffered 3.38 million confirmed cases and 81,228 fatalities.

In fact, despite issues of inadequate medical resources and preparedness, the official response to the pandemic in African countries such as Ghana has been quite timeous and progressive [7]. These measures so far have included lockdowns in high-risk areas, isolation of infected persons and contact tracing, advocating personal preventive practices, such as wearing face masks, using alcohol-based sanitizers, hand washing with soap, and social distancing [8,9]. Testing facilities have been in place early in Ghana and an infectious disease center has been built in the capital city Accra and completed during the ongoing COVID-19 pandemic [10].

In parallel, public health campaigns to curb the spread of the ongoing COVID-19 pandemic have been in place. The knowledge of the population with regard to the nature, symptoms, transmission, and prevention of an infectious disease is often considered pivotal in influencing the attitudes and practices towards tackling a pandemic. Nonetheless, knowledge alone may not suffice to convince the population to implement preventive measures or to become vaccinated as some of the recent shocking news from across Europe have demonstrated [11,12,13,14]. 

We have therefore carried out a cross-sectional study to characterize the knowledge of the Ghanaian population related to COVID-19 and to investigate the postulated link between this knowledge on the one side and the implementation of preventive measures in practice on the other. Such cross-sectional studies reporting the knowledge and practice of different populations are conducted routinely to evaluate and better design public health campaigns and interventions [15]. In this context, we also have elucidated whether there are any socio-demographic factors influencing either the knowledge or compliance with the preventive measures. One may, for instance, expect that higher levels of knowledge related to COVID-19 along with certain socio-demographic factors such as age, education, occupation and/or type of community, may be valuable determinants of better compliance with preventive measures including personal hygiene and willingness to become vaccinated.

## 2. Materials and Methods

### 2.1. Preparation of the Questionnaire

To address these issues of information about COVID-19 and implementation of measures against it among the wider population across Ghana, a questionnaire was developed, pretested, and validated as shown in Supplementary-Section S5. The questionnaire was also translated from English to local languages of participating communities, including Twi, Ewe, Ga, Gonja, Kussal, and Buli by professional linguists for the few participants who could not understand English. Their answers were translated back to English. In the field, the principal investigator and trained research assistants then read the 26-item questionnaire to the respondents and collected their answers.

As for the questionnaire itself, it is divided into three main sections, namely, socio-demographics, knowledge, and practice of preventive measures. The socio-demographics collects information on age, gender, marital status, educational level, occupation, religion, and type of community. The knowledge section consists of four subsections. The first subsection has two categorical items addressing general knowledge of infectious diseases. The following two subsections address the knowledge of the respondents on cholera and influenza. The last subsection assesses the knowledge of the respondents of COVID-19 with nine categorical items. By the term knowledge we aim to capture the facts, awareness, or information obtained through experience or education related to the COVID-19 pandemic. The WHO recommendations on preventive practices to stop the spread of COVID-19 are used as a standard in evaluating the practices of the respondents, such as wearing a face mask, practicing social distancing, regular hand washing with soap, and willingness to receive the vaccine.

It should be noted that the topics of the questionnaire are highly specialized, ranging from knowledge of infectious diseases, symptoms, and modes of transmission to different preventive practices. These questions are addressed to the broad public excluding healthcare professionals.

### 2.2. Study Design and Data Collection

**Figure 1:**
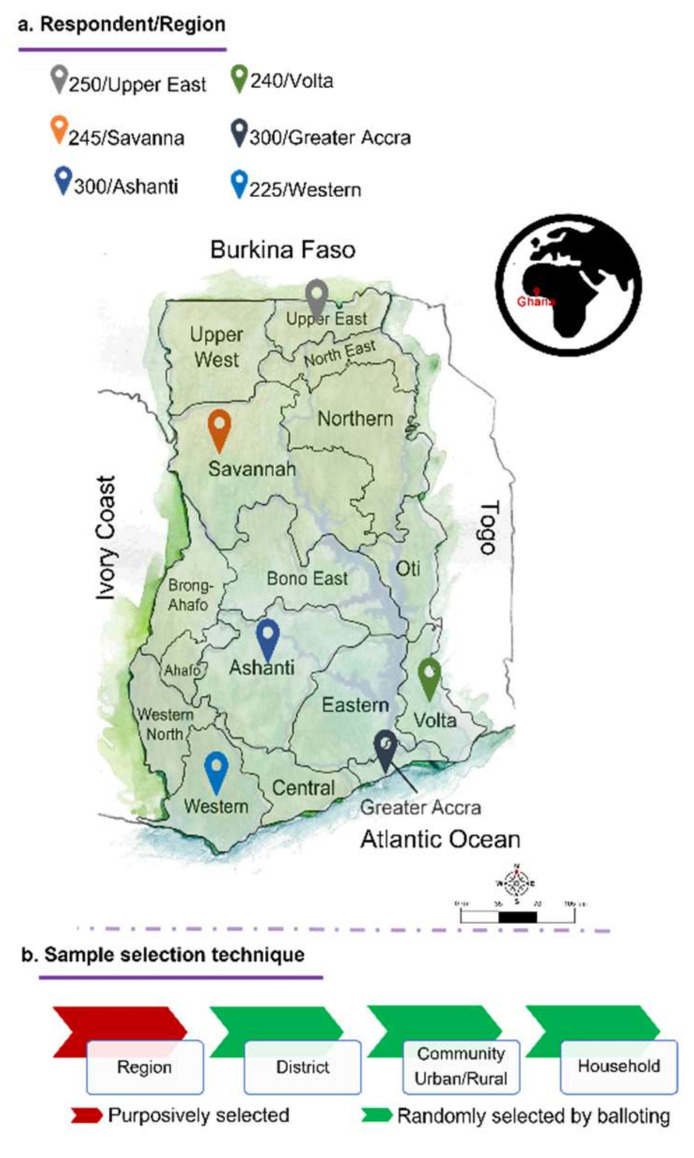
(**a**) Geographic locations of sampling within Ghana on the right and, (**b**) a scheme of sampling technique (sketch provided by Leja Nessis).

The cross-sectional study employing an interview-structured questionnaires (*n* = 1560) was carried out in six different regions of Ghana, including Upper East, Savannah, Ashanti, Western, Greater Accra, and Volta regions, Figure 1a.

The study followed a multistage sampling technique, Figure 1b. The six regions for the study were selected to represent the northern, middle, south-western, and south-eastern parts of the sixteen regions in Ghana. On the district, community, and household level the sampling technique was randomized. The study was carried out from September 2020 to December 2020. Data collection was completed on Saturdays and Sundays as most respondents should be at home then. During sampling, the interviewers and the participants adhered to the COVID-19 preventive measures.

Health-care workers were excluded as they are likely to be trained in disease-related countermeasures. As the study was based on an anonymous survey and did not include human or animal specimens, ethical clearance was addressed to the relevant authorities and obtained from the Ethics Committee on Human Research Publication and Ethics, School of Medical Sciences, KNUST Kumasi, Ghana. Moreover, informed consent forms were obtained from each of the respondents. In the case of participants below 18 years of age, consent was sought from their guardians. The questionnaires were completed by these volunteers and collected on the spot, checked manually for completeness, and then taken to Kwame Nkrumah University of Science and Technology (KNUST) for processing.

### 2.3. Statistical Procedure

The raw data were entered in Microsoft Excel version 16.34 (2019) where data cleaning, validation, and quality checks were carried out. The analysis of the data employed linear multiple regression models and was conducted using *R* (*R* core team, 2021) including car [16] and boot packages [17] at the Department for Economics and Sociology of Sports, Faculty of Economics and Empirical Human Sciences, Saarland University, Germany.

The data were tested (Chi-squared test) for representativeness of both the regional and overall population in Ghana, Supplementary Section S6. The sample in this study was found to be not representative, as a result, descriptive statistics were calculated as weighted means. The weights were calculated from the univariate marginal distributions, thus assuming that age, gender, and region were independent. Therefore, age, gender, and region were included in the statistical models as instrumental variables (IV) to control for error variance and confounded results from unequal cross-distributions, see Supplementary Section S1.

As for the linear multiple regression analyses, gender was reduced to binary (male/female), region was dummy-coded to provide significance tests for contrasts between each region and the capital region Accra and age was recoded into three groups (15–24), (25–54), and (55+), and was dummy-coded to provide significance between the younger group and the two older groups. The remaining sample for the regression analysis was *n* = 1516. Only weak multicollinearity assessed via the intercorrelation matrix models and the variance inflation factor VIF, was observed throughout. The Shapiro–Wilk Test and also skewness and kurtosis were assessed for normality in the distributions of variables. In the case of deviation from normality, a bias-corrected and accelerated bootstrap interval, and 2000 bootstrap-replications of the designated regression coefficients were computed. *t*-values, *beta* coefficients, and adjusted *R*-squared values were reported, as appropriate. A test for the independence of errors (Durbin–Watson Test), outliers and inferential cases, and one-sided 5% Bootstrapped Confidence intervals were reported when applicable, see Supplementary Section S2.

Scores weighted to represent the sample at the regional level are reported, when applicable, are provided in Supplementary Sections S2–S4. The scores were calculated via the mean number of correctly ticked answers and correctly unticked answers, and hence a higher accuracy of the measurement was achieved due to the higher number of items per domain.

## 3. Results

In general, the data obtained confirm that there is a good level of knowledge within the communities in Ghana about the SARS-CoV-2 infection and the pandemic caused by this virus. This knowledge of COVID-19 may indeed be the result of a general alertness due to other outbreaks having affected Ghana in the past (influenza and cholera). Despite this level of knowledge, there was a certain low willingness to comply with the COVID-19 preventive practices/measures to counteract the pandemic and also a low inclination for vaccination. Furthermore, though the linear multiple regression analysis demonstrated that the knowledge of COVID-19 associated positively with the practice of preventive measures, the level of explained variance of the model was as low as 6.9%. There was no substantial influence of any of the socio-demographic characteristics surveyed on the practice of the preventive measures.

In what follows, we first present the descriptive statistics of the sample. In the knowledge and practice sections, the results are presented as weighted mean numbers and standard deviations of the scores from our sample to represent the Ghanaian population. 

### 3.1. Descriptive Statistics

#### 3.1.1. Socio-Demographic Indicators

**Figure 2:**
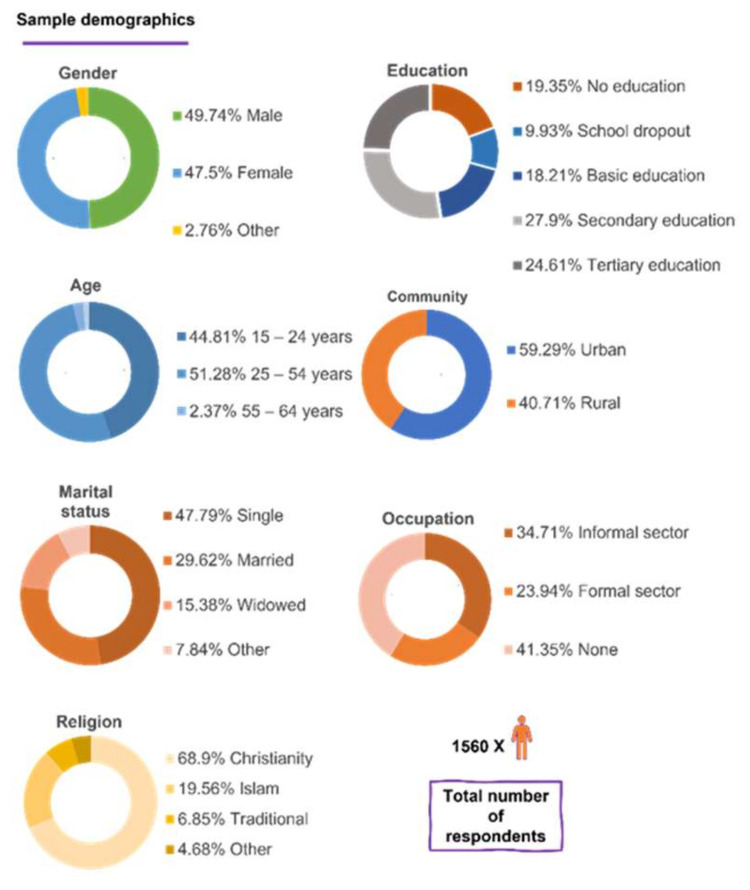
The socio-demographic characteristics of the 1560 respondents of the study.

The socio-demographic characteristics of the 1560 respondents in the survey and regional data are summarized in Figure 2.

#### 3.1.2. Data Obtained from the Knowledge Section

The scores of the population sampled in the knowledge section are presented as weighted mean numbers and standard deviations in Table 1.

The average result in the general knowledge of infectious diseases was 65.4%. The average knowledge scores of COVID-19 obtained were also close to 70%, implying that many respondents questioned held an adequate level of knowledge about infectious diseases and the COVID-19 pandemic. An average summative score of 67.1% was obtained in the knowledge section of the questionnaire, as shown in Supplementary Section S1.

#### 3.1.3. Data Obtained from the Practice Section

Only about 31% of the estimated population indicated positive responses to the practice of preventive public health measures. With regards to vaccination against COVID-19, which has been on the roll-out in Ghana since March 2021, only around 35% of the population showed willingness towards getting vaccinated, Table 2.

### 3.2. Linear Multiple Regression Analysis

#### 3.2.1. Influences on the Knowledge of COVID-19

**Figure 3:**
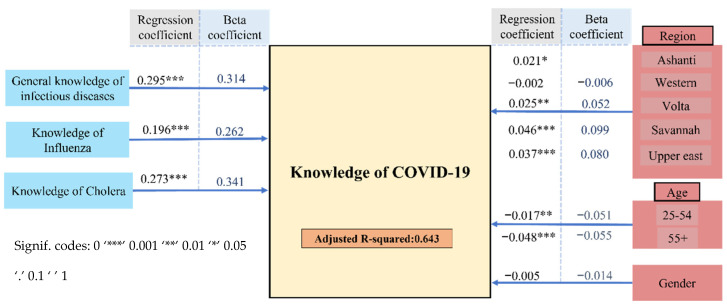
The knowledge of COVID-19 Model (KCM) which was employed to assess the influences of the other domains of knowledge surveyed on the knowledge related to COVID-19. The results are shown in terms of regression coefficient estimates (non-standardized coefficient) and *beta* coefficients (standardized coefficient).

From the scores obtained in the knowledge section, one may hypothesize that prior knowledge on previous outbreaks other than COVID-19, such as cholera and influenza, may sensitize the population for pandemics. Figure 3 indicates significant positive associations between having good general knowledge of infectious diseases along with more specific knowledge of existing infectious diseases, cholera, and influenza, on the one side and knowledge of COVID-19 on the other.

The values of the regression coefficient (unstandardized coefficient) and *beta* coefficient (standardized coefficient), which have values ranging from −1 and +1, represent the mean change in the response given a one unit change in the predictor. So, for instance, an increase of one unit in the respondent’s knowledge related to cholera, would lead to an increase of 0.273 in their knowledge related to COVID-19. However, when the variance between their knowledge related to COVID-19 and cholera is standardized, the increase would be 0.341. Furthermore, the adjusted R-square value for this model was 0.643. This means that the difference in the knowledge of COVID-19 among the population can be explained by their knowledge related to influenza and cholera while considering age, gender, and region by a level of more than 64%.

**Figure 4:**
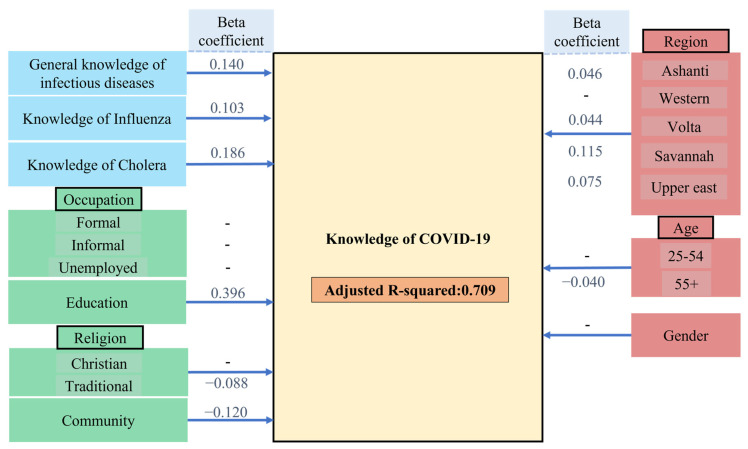
The extended knowledge of COVID-19 model (E-KCM) employed to assess the influences of the socio-demographic characteristics on the knowledge of COVID-19. The results are shown in terms of regression coefficient estimates and *beta* coefficients. The level of explained variance in the results of this model was 70.9%.

The model was extended stepwise to assess for the influence of other socio-demographic characteristics of the sample. The type of community, whether rural or urban, affected the variance in the knowledge of COVID-19 variable only slightly, and the explained variance rose insignificantly from 64.4% to 65.4%. As anticipated, education and occupation also increased the explained difference in knowledge of COVID-19 from 65.4% to 70.3%, which is significant at *p* = 0.001, implying a significant influence of education and occupation on knowledge of COVID-19. Apart from that, accounting for religious differences slightly influenced knowledge of COVID-19, as it increased the level of explained variance by 0.6%; final adjusted R-squared = 0.709, Figure 4, Supplementary Section S3.

It should be noted that in this model, the Akaike information criteria (AIC) demonstrated that education—given the non-significant contribution of occupation—adds substantially to the explanation of the differences in the knowledge of COVID-19 among the respondents, Supplementary Section S3.

#### 3.2.2. Knowledge onto Practice

**Figure 5:**
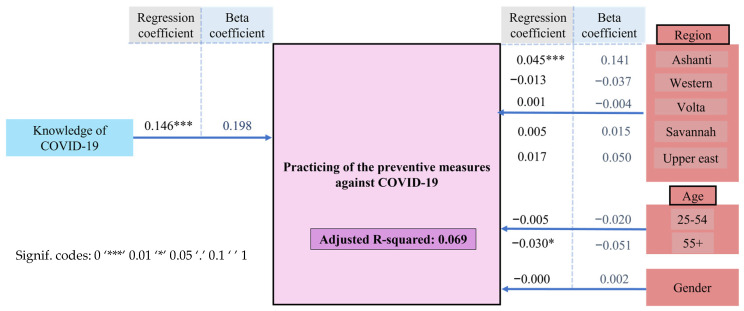
The practices against COVID-19 model employed to assess the influences of knowledge related to COVID-19 and the practicing of preventive measures recommended by the WHO. The results are shown in terms of regression coefficient estimates and *beta* coefficients.

A significant although weak association was also found between the knowledge of COVID-19 and the practice of preventive measures against it, Figure 5.

Unexpectedly, the linear regression model explicated just 6.9% (adjusted *R*-square = 0.069) of the relationship between the level of knowledge related to COVID-19 and the low compliance with preventive measures. This implied no substantial influence of knowing about COVID-19 and practicing of preventive measures, Supplementary, Section S4.

This model was extended by stepwise addition to assess the influence of the type of community, level of education, occupation, and religion of the respondents on their practice of preventive measures against COVID-19. Being employed in the formal sector significantly associated with better practice but comparing the AICs of the models showed that the increase in explanatory power was nearly neutralized by the increased complexity of the model. The Bayesian information criterion (BICs), showed that the more complex model would be classified as worse than the basic model. In other words, none of the socio-demographic characteristics meaningfully changed the level of explained variance of the practice of the respondents against COVID-19, ultimately contributing just 3.7% of the level of explained variance. This indicates that there was no substantial influence of either gender, age, religion, education, occupation and, indeed, the type of community one resides in, on the practice towards preventing the SARS-CoV-2 infection and the pandemic as detailed in the statistical model, Supplementary Section S4.

## 4. Discussion

In general, the data suggest that in Ghana the campaign to inform the wider population about the SARS-CoV-2 pandemic and its risks has been successful. This campaign has reached virtually every sector of the Ghanaian society, in bigger cities and also in the remote countryside. Although this is very encouraging, the limited readiness of the same individuals to protect themselves, for instance, by wearing face masks, washing hands with soap, or maintaining social distancing is worrisome and may require further action, especially in the context of the vaccination program. These issues are now discussed in more detail.

Firstly, our study suggests that public knowledge of COVID-19 in Ghana is generally good, as the overwhelming majority of the 1560 respondents questioned, in cities and also in the countryside scored an average of 69.9%. These results are indeed comparable to studies from neighboring countries such as Côte d’Ivoire, Burkina Faso, Nigeria, Uganda, and Gambia [18,19,20]. These findings may be explained with prior exposure and experiences of the population to and with similar infectious outbreaks, for instance, cholera, which accounts for 3.8% of fatalities in Ghana each year [21]. Indeed, knowledge of COVID-19 at 69.9% was associated positively with knowledge of other existing infectious diseases—cholera and influenza, with a 64.3% level of explained variance among the population. Knowledge related to these two infectious diseases, along with the socio-demographics surveyed accounted to more than 70% of the difference among the knowledge of the population about COVID-19, Figure 3 and Figure 4.

Secondly, and this may be the most astonishing finding of the entire study, this generally adequate knowledge does not translate into a general compliance with the personal preventive public health measures. These basic preventive measures are only followed by around 30% of the population, as presented in Table 2. Knowledge related to COVID-19, being of a younger age and living in the capital city Accra associated positively with practicing the preventive measures. Nonetheless, the differences among the population, even after including further socio-demographic variables, remained indeed high and were barely explained by the regression model presented in Figure 5 and Supplementary Section S4. Therefore, the question of why the population is not practicing the preventive measures remains open. Here, an interplay between economic, social, psychological, and environmental structural factors, operating on individuals and also on groups, might be considered to be hindering acceptability, accessibility and availability of such preventive measures in Ghana [22,23]. In this sense, the effect of knowledge onto practice could be moderated by other factors beyond the scope of this study [24,25,26].

In our study, for instance, only about 29% of the respondents were willing to wear a face mask. While the individual daily average living wage in Ghana is around GHC 30 (USD 4.89), locally produced face masks cost between GHC1 (USD 0.17) and GHC 7 (USD 1.20). In contrast, the price of N95 masks is around GHC 25 (USD 4.37) per mask [27,28]. Since wearing a face mask on public transportation was rendered mandatory in Ghana, in April 2020, the accumulating costs, amplified, perhaps, by low perceived benefits of the face mask on a hot day in public transport, may indeed lower the acceptability of face masks [28]. In the same breath, unwillingness to regular hand washing can also be viewed as a consequence of the customs and traditions of the population [29]. Notwithstanding, the *Veronica bucket*, a revolutionary, simple and local invention which became popular in 2020 in response to the pandemic and has rendered washing hands with soap and water available to the population at many public places [30]. It should also be noted that the collective and inclusive social structures in Ghana, such as the crowded local, informal markets, in which the majority of the population earn and shop, added to the household structure, which is reported to be congested with individuals of varying ages could also hamper the compliance with the preventive measures [8,31].

Furthermore, investigating and addressing psychological factors on the attitudinal level, such as stigma, self-efficacy, group-efficacy, perceived severity of disease, and perceived barriers could also explain the discrepancy between knowledge on the one side and individual attitude and adeptness to follow the preventive measures and vaccination on the other [32,33,34]. Several such structural factors, belonging to different settings and contexts have been addressed as part of this special issue, Reducing Health Inequities: Social Epidemiology Insights for Public Health and Social Policy [35,36,37,38,39]. 

**Figure 6:**
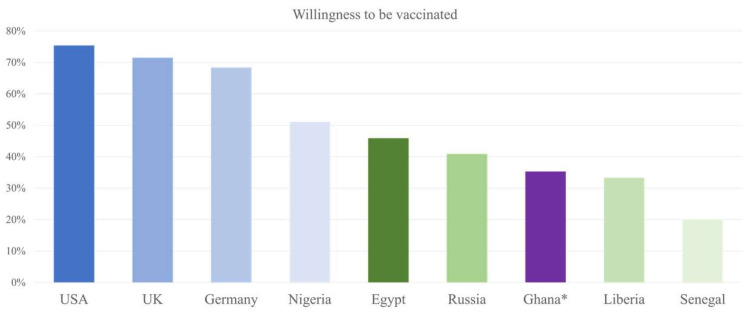
The readiness to receive vaccines against COVID-19 in different countries compared to Ghana. * Results reported in our study.

In the case of willingness to receive the vaccination, our findings agree with the ones provided by the so-called Afro barometer survey conducted in March 2021 in five African countries, namely Benin, Liberia, Niger, Senegal, and Togo. The survey reported that only 4 in 10 respondents (*n* = 1200) were likely to take the vaccine [40]. The rates of willingness to be vaccinated presented in Figure 6 indeed elicit wider issues with compliance in many countries in Africa and globally which do not necessarily simply rely on sufficient information about the disease and the countermeasures in place [40,41,42]. In fact, misinformation about the COVID-19 vaccines and their benefits is common, especially on social media, and has already resulted in hesitancy towards vaccination [43,44]. In fact, one of our previous studies conducted in Ghana has already documented a cultural element regarding the choice of antimalarial drugs [45]. In this study, the recommendation by friends guided the choices of 93.6% of the respondents in Ghana on the preference of herbal medicines used against malaria. In any case, these findings also define a potentially major problem for the next couple of years, as producing and providing vaccines alone may not be sufficient unless there is a general willingness to be vaccinated.

Indeed, whilst developed countries such as Austria, Germany, and the Netherlands should be close to achieving protection by vaccination, countries such as Ghana, where only about 3% of the population is fully vaccinated to date might allow COVID-19 to linger on and also to produce new and more dangerous variants [44,45,46]. This may not only be due to a lack of availability of vaccines but also the acceptance from the population. The willingness of the respondents to be vaccinated resided at 35.3%, which might indicate that availability of the vaccine alone might not be the problem. For instance, some individuals may distrust the sources of the vaccines and develop fear of possible side effects. Here, remembering the deleterious Tuskegee study of untreated syphilis in African American males in 1932 may contribute to the reluctance [47]. The prognosis of failing to wisely promote the vaccines could lead to a huge humanitarian crisis on an international scale [48].

Africa and indeed the world at large, may therefore provide new hotspots of new variants of the virus as, indeed, has been the case with the new variants isolated in South Africa, Brazil, and, recently, India [45,49]. As this low level of compliance is almost independent of socio-demographic indicators, such as gender, age, education, occupation, religion, community, and region, simply running more public campaigns in different groups of the population and different areas of Ghana may not resolve this problem. Additional approaches should include a consistent and proper communication between the public and governmental bodies involved in the procurement of the vaccine [41]. The recent experience of Ebola vaccination across several African countries demonstrates strong social and political hesitancy to the introduction of new vaccines [40,43,45,46,48,50]. Campaigns of public awareness and interpersonal engagements involving health practitioners and trained vaccination assistants from and within the communities should be put in place and encouraged. This may help achieve an incremental uptake of the vaccine in the country, as aimed at by the government of Ghana. Furthermore, household elders, traditional, religious, academics and other opinion leaders, in modern English often referred to as “social influencers”, may serve as channels of communication, in public and also in the social media. Vaccinating these formal and informal leaders and influencers, perhaps in the open as often exercised by politicians, may help to relieve and to reduce the skepticism. As for scientists, it is imperative for Ghana, Africa, and the global community to study the challenges with compliance in order to put measures in place, as willingness to become vaccinated, rather than availability of a suitable vaccine, may soon become a major obstacle in the fight against the virus.

## 5. Conclusions

To conclude, our study involving respondents from various subgroups of, and locations in, Ghana confirms that the population is by large properly informed about the SARS-CoV-2 pandemic. Nonetheless, compliance with preventive measures and willingness to take the vaccine is low. We could not find any meaningful correlations between this low readiness to follow the precautionary measures on the one side and common socio-demographic parameters on the other. The manifestation of this mismatch between knowing about COVID-19 and non-complying with its preventive measures is not limited to Ghana, as major demonstrations against COVID-19 measures and vaccination on the streets of major European countries, such as Amsterdam, Berlin, and Vienna, demonstrate [11,12,13,14,51,52]. Indeed, attempts to enforce preventive measures and vaccinations by the governments in these countries already have led to riots and political unrest. In fact, some of the findings of our study conducted in the second half of 2020 have already heralded these developments, including a perhaps initially suspected resistance of the virus against the vaccines and its ability to result in further outbreaks and more dangerous variants, such as the Omicron variant. It is therefore pivotal to discuss and address additional factors such psychological, social, political, economic, and environmental attitudes and influences.

Considering these rather distressing findings of our present study together, effective measures need to be put in place to convince the population in Ghana of the necessity and benefits of adopting precautionary measures, including vaccination against the virus. Therefore, once vaccines become available more widely, a campaign must be in place to convince and educate the wider population to the benefits of these vaccines. This task may be shouldered by the government and should be supported by the traditional and modern influencers on the ground, in public, and possibly also on social media. A qualitative study to unravel why, despite the adequate level of knowledge on infectious diseases and COVID-19, only a few individuals are willing to take precautionary measures and be vaccinated should be carried out to drive policy directions. Informed policy, would indeed avoid unrest, similar to what occurred in Europe, and to avoid turning Africa into the home of new and perhaps more dangerous variants.

## Figures and Tables

**Table 1:** The level of knowledge of the Ghanaian population on existing infectious diseases and COVID-19.

	Mean	SD
General Knowledge of infectious diseases	0.654	0.188
Influenza	0.579	0.240
Cholera	0.752	0.222
COVID-19	0.699	0.190
Summative score	0.671	0.177

**Table 2:** Responses to the different preventive practices/measures presented as average weighted mean numbers.

Preventive Practice	Response (%) Yes
Wearing of face mask	28.7
Regular hand washing	31.7
Social distancing	28.0
Willingness to be vaccinated	35.3

## Data Availability

Data is available upon request.

## Supplementary Materials

The following are available online at https://www.mdpi.com/article/10.3390/ijerph182412902/s1, Supplementary Section S1: Descriptive statistics, Section S2: Inferential statistics, Section S3: Linear Multiple Regression Analysis: Influences on the Knowledge of COVID-19, Section S4: Linear Multiple Regression Analysis: Influences on the practice against COVID-19, Section S5: Questionnaire, Section S6: Test for Representativeness.
