# Supplementary material for: Knowledge into the Practice against COVID-19: A Cross-Sectional Study from Ghana
Source: Int J Environ Res Public Health. 2021 Dec 7;18(24):12902. doi: 10.3390/ijerph182412902 (PMC8702109; doi:10.3390/ijerph182412902)
Supplement: Supplementary file 1 [file ijerph-18-12902-s001.zip › ijerph-1422591-supplementary.pdf]

Supplementary Material

Knowledge into the Practice against COVID-19: A Cross-sectional Study from Ghana

Section S1: Descriptive statistics

1.1 Sociodemographic frequencies of the respondents

AGE	n	%
15-24	699	44.81
25-54	800	51.28
55-64	37	2.37
65+	23	1.47

GENDER	n	%
MALE	776	49.74
FEMALE	741	47.50
OTHERS	43	2.76

Calculated using function xtabs and norming the results to the sample size rounded to zero digits as well as in percentages. Code example for Cross tabulation Religion * Gender:
One-dimensional frequency distributions, corrected for representativeness for those variables which were not used for correction:

Religion	Christianity	Islam	Traditional	None
	1043 (68.90%)	296 (19.56%)	104 (6.85%)	71 (4.68%)

Education	No education	School dropout	Basic education	Secondary education	Tertiary education
	293 (19.35%)	150 (9.93%)	276 (18.21%)	422 (27.90%),	373 (24.61%)

Occupation	Informal sector	Formal sector	None
	526 (34.71%),	362 (23.94%),	626 (41.35%)

Marital status	Single	Married	Divorced	Widowed	prefer not to say
	724 (47.79%)	448 (29.62%)	46 (3.07%)	233 (15.38%)	63 (4.14%)

Community	Urban	Rural
	898 (59.29%)	616 (40.71%)

Regional	Greater Accra	Ashanti	Western	Volta	Savanna	Upper East	Total
	300 (310 sent)	300	225	240	245	250	1560

1.2 Knowledge Scores

	Mean	SD
General Knowledge of infectious diseases	0.654	0.188
knowledge Influenza	0.579	0.240
knowledge of cholera	0.752	0.222
Knowledge of COVID-19	0.699	0.190
Existing Infectious Diseases	0.662	0.182
Existing Infectious Diseases + COVID-19	0.671	0.177

1.3 Practices Scores

Weighted for representativeness in the studied regions:

```
for(i in 1:length(varlist)){
+   print(colnames(ID_weighted[varlist[i]]))
+   tab <- xtabs(ID_weighted$weight ~ unlist(ID_weighted[, c(varlist[i])]))
+   print("frequency table")
+   print(round((tab * nrow(ID_weighted[ID_weighted$weight > 0, ]) / sum(tab)), 0))
+   print("percentage")
+   print(round((tab * 100 / sum(tab)), 2))
+ }
```

Weighted for representativeness for the whole population:

```
> # creating weighted frequency tables for the practice items
> varlist <-c(90:97,99:102)
> for(i in 1:length(varlist)){
+   print(colnames(ID_weighted_population[varlist[i]]))
+   tab <- xtabs(ID_weighted_population$weight ~ unlist(ID_weighted_population[, c(varlist[i])]))
+   print("frequency table")
+   print(round((tab * nrow(ID_weighted_population[ID_weighted_population$weight > 0, ]) / sum(tab)),
0))
+   print("percentage")
```

+   print round((tab * 100 / sum(tab)), 2))

Assessment of practices against COVID-19

Pertinent practices to prevent COVID-19 spread	Response			
	Yes		No	
	Studied Region (freg) %	Whole population (freq) %	Studied Region (freq) %	Whole population (freq) %
Wearing nose mask	(426) 28.14	(435) 28.73	(1088) 71.86	(1081) 71.27
Regular hand washing	(525) 34.7	(481) 31.74	(989) 65.3	(1035) 68.26
Social distancing	(408) 26.93	(425) 28.06	(1106) 73.07	(1091) 71.94
Willingness to take vaccination	(516) 43.06	(535) 35.31	(998) 65.94	(981) 64.69

Section S2: Inferential statistics

2.1 Initial Tests for Assumptions

For the inferential analyses, region, gender and age group were always included as IV's in order to reduce error variance and not to have the results confounded by the differing distributions. Therefore, basic assumptions on these variables were assessed first:

2.1.1 Variable types:

For gender, the level "prefer not to say" is omitted from the analysis. The remaining factor is binary.

Region and age group are categorial and need being transferred into contrasts:

- Region was dummy-coded to provide significance tests for contrasts between each region and the capital region Accra.
- Age group was be recoded into three age groups 15- 24, 25 – 54, 55 – in order to avoid cells with zero frequencies in the design. The remaining three level factor was dummy-coded by the function contr. treatment with base= "15-24" meaning that significant effects will be calculated for contrasts between the other two age groups and the base age group.

With these recordings, 1516 records remain for regression analyses.

2.1.2 Multicollinearity:

Multicollinearity for gender, age group and region were studied basing on the intercorrelation matrix as well as for individual regression analysis on the VIF.

The (Spearman) intercorrelation matrix showed only marginal correlations

	Gender	Region/Area	agegroup
Gender	1.000	0.039	-0.006
Region/Area	0.039	1.000	-0.102
age group	-0.006	-0.102	1.000

2.1.3 Knowledge variables, operationalized via the mean number of correct answers:

Testing assumptions on further model variables.

2.1.3.1 Test for normality

overall:

	KNOWLEDGE General	Knowledge Influenza	Knowledge Cholera	Knowledge COVID-19	Knowledge ID	Knowledge IDCOVID-19
median	0.667	0.667	0.875	0.778	0.727	0.737
mean	0.675	0.632	0.772	0.731	0.693	0.702
skewness	-0.561	-0.306	-1.195	-1.064	-0.729	-0.832
skew.2SE	-4.458	-2.427	-9.504	-8.452	-5.782	-6.596
kurtosis	0.186	-1.169	1.044	0.543	-0.103	-0.024
kurt.2SE	0.739	-4.646	4.154	2.159	-0.410	-0.095
Normtest.W	0.942	0.928	0.857	0.904	0.951	0.934

P	< 0.001	< 0.001	< 0.001	< 0.001	< 0.001	< 0.001

Comparing means and medians show that - given the normed maximum range of 1 - most variables are more or less symmetrically distributed. Nevertheless, skewness and kurtosis (should be zero if normality would be given, absolute values of skew.2SE and kurt.2SE should be < 1) differ significantly from normality. This is also shown in the significant Shapiro-Wilks test (2 lower lines). Double-checking this for a smaller random sample from the dataset (n=100) to avoid over-petty testing due to extremely large sample size:

	Knowledge General	Knowledge Influenza	Knowledge Cholera	Knowledge COVID-19	Knowledge ID	Knowledge IDCOVID-19
skewness	-0.372	-0.225	-1.406	-0.957	-0.509	-0.691
skew.2SE	-0.771	-0.466	-2.913	-1.982	-1.055	-1.431
kurtosis	-0.118	-1.457	2.075	0.334.	-0.764	-0.453
kurt.2SE	-0.124	-1.523	2.169	0.350	-0.799	-0.474
Normtest.W	0.938	0.896	0.843	0.917	0.951	0.936
Normtest.P	0.000	0.000	0.000	0.000	0.001	0.000

Also, with the smaller sample, skewness for most of the variables differed significantly from normality as was also indicated by the Shapiro-Wilks-Test. Comparisons between q-q norm-plots and the ideal q-q lines indicated that differences from normality were not merely trivial (as e.g. because of the limited accuracy of measurement). For knowledge scores based on mean correct answers, there were significant deviations from normality. As a result, significance of coefficients in regression models including knowledge variables were secured by bootstrapping.

2.1.3.2 Intercorrelations/multicollinearity

Domain-specific knowledge variables (general, influenza, cholera and COVID-19) correlated between 0.457 and 0.637. Therefore, there was no strong multicollinearity assumed. Nevertheless, for analyses which covered these variables as independent variables, multicollinearity was additionally tested for each individual model.

	Knowledge General	Knowledge Influenza	Knowledge Cholera	Knowledge COVID-19	Knowledge ID	Knowledge IDCOVID-19
Knowledge General	1.000	0.537	0.548	0.637	0.812	0.808
Knowledge Influenza	0.537	1.000	0.457	0.600	0.851	0.835
Knowledge Cholera	0.548	0.457	1.000	0.620	0.770	0.767
Knowledge COVID-19	0.637	0.600	0.620	1.000	0.736	0.828
Knowledge Infectuous diseases	0.812	0.851	0.770	0.736	1.000	0.987

Knowledge Infectuous diseases including COVID-19	0.808	0.835	0.767	0.828	0.987	1.000

2.1.3.3 Practices, normality assumption

	Practices, whole sample	Practices, random subsample (n=100)
median	0.333	0.333
mean	0.314	0.328
skewness	0.263	0.012
skew.2SE	2.118	0.024
kurtosis	-0.176	-0.195
kurt.2SE	-0.709	-0.203
Normtest.W	0.959	0.950
p	< 0.001	< 0.001

The median was close to the mean, indicating a nearly symmetric distribution. Nevertheless, the skewness significantly differed from the skewness of a normal distribution. While kurtosis did not, the Shapiro-Wilks-test yielded a significant result. Likewise, the knowledge scores, this was double checked using a smaller random sample (n= 100). For this sample, the Shapiro-Wilks-test also showed a significant deviation from normality which was very likely due to the limited accuracy of measurement, as is shown in the following q-q-plot.

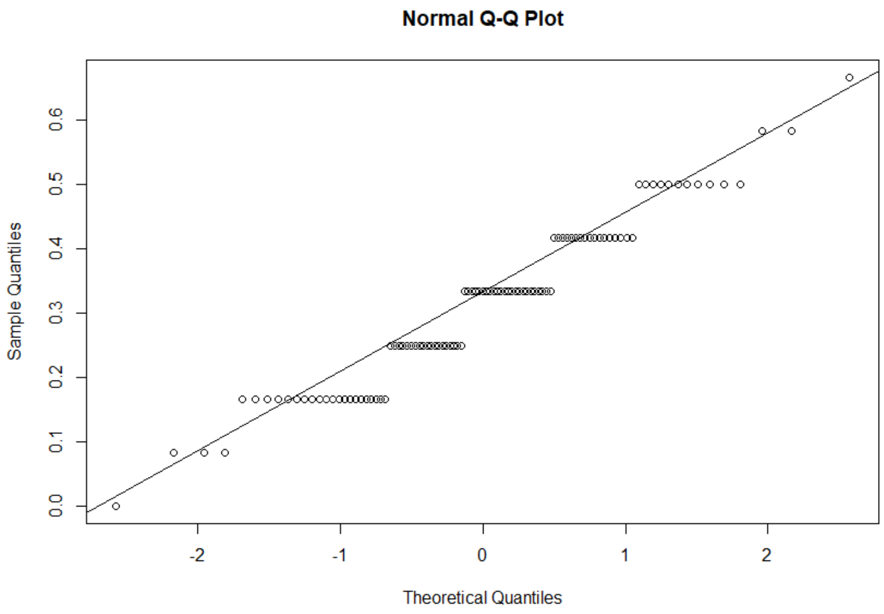

As a result, Regression analyses results were secured by Bootstrapping.

2.2 Extending the knowledge and practice models

The Knowledge of COVID-19 and the Practices against COVID-19 Models, presented later in sections A4 and A5, were extended stepwise to include further sociodemographic characteristics. This led to 3 additional steps in the model, first community (urban vs. rural), then education and occupation and at last, religion were added to the models. As the community was only measured binary, this was added as-is to the models. Education was added as a linear factor with increasing figures indicating a higher level of education. Occupation was measured at the three levels “formal sector”,” informal sector” and “none” Therefore, occupation was entered via contrasts, using the category “none” as base category.

```
ID_comparison$Occ_factor <- as.factor(ID_comparison$Occupation)
ftable(ID_comparison$Occ_factor)
  0     1       2

 512   406     577
contrasts(ID_comparison$Occ_factor) <- contr.treatment(seq(0:2), base = 3)
```

Due to the uneven distribution of religions (see below) and the low frequencies in cat. 2 (“Traditional”) and 3 (“none”), this variable was reduced to two levels “Christian” vs. “non-Christian”

```
  0     1       2       3

 1015  364     68      48

ID_comparison$Religion_factor <- ID_comparison$Religion
RepVector <- ID_comparison$Religion > 0
ID_comparison [RepVector, 19] <- 1
```

Section S3: Linear Multiple Regression Analysis: Influences on the Knowledge of COVID-19

Reformulated to: Does the level of knowledge of existing infectious diseases correlate to the level of knowledge, and to practice/attitude towards COVID-19 ? What are the influential sociodemographic characteristics in this context?

3.1 Model formulation for DV knowledge on COVID-19

The knowledge of COVID-19 Model was built to identify the influence of domain-specific knowledge on COVID-19- knowledge. Age, gender and region included to control for error variance and confounded results from inequal cross-distributions. Bootstrapping with bca-corrected CI's and 2000 Bootstrap-replications

Call:
lm (formula = Knowledge COVID-19 ~ Knowledge General + Knowledge Influenza + Knowledge Cholera +
age factor + region factor + Gender, data = ID_regression)

Residuals:
Min 1Q Median 3Q Max
-0.37252 -0.06391 0.008330.067440.35765

Coefficients:	Estimate	Std.	Error	t value	Pr(>|t|)	Bootstrapped 5% conf-limit
(Intercept)	0.188109	0.013542	13.890	<2e-16	***	0.1657
General knowledge of infectious diseases	0.295620	0.019675	15.025	<2e-16	***	0.2608
Knowledge of Influenza	0.196273	0.014920	13.155	<2e-16	***	0.1693
Knowledge of Cholera	0.273464	0.015934	17.162	<2e-16	***	0.2448
Age 25-54	-0.017520	0.005630	-3.112	0.001894	**	-0.0085
55+	-0.048992	0.014377	-3.408	0.000673	***	-0.0169
Ashanti	0.021618	0.008899	2.429	0.015250	*	0.0072
Western	-0.002893	0.009644	-0.300	0.764210		n.s.
Volta	0.025468	0.009296	2.740	0.006224	**	0.0076
Savannah	0.046547	0.009256	5.029	5.53e-07	***	0.0313
Upper east	0.037893	0.009609	3.943	8.40e-	***	0.0212

				05		
Gender	-0.005126	0.005377	-0.953	0.34063 0		n.s.

Significance. codes: 0 ‘***’ 0.001 ‘**’ 0.01 ‘*’ 0.05 ‘.’ 0.1 ‘’ 1

Residual standard error: 0.1038 on 1494 degrees of freedom
(10 observations deleted due to missingness)
Multiple R-squared: 0.6462, Adjusted R-squared: **0.6436**
F-statistic: 248 on 11 and 1494 DF, p-value: < 2.2e-16

The one-sided 5% Bootstrapped Confidence intervals led to the same statistical decisions as the t-Test did. Therefore, the alpha-error probabilities can well be assumed to be not heavily biased by the violation of the assumption of normality.

The model explained 64.36 % of the variance of the dependent COVID-19 knowledge variable.

This Model was compared to a Basic Model, including only age, gender and region as independent variable. The basic model, although significant, explained only 8.97 % (adjusted R^2) of the variance of the dependent variable. The models differed significantly.

Anova (BasicModel_Knowledge, COVID-19Model_Knowledge)
Analysis of Variance Table

Model 1: Knowledge COVID-19 ~ age_factor + region_factor + Gender
Model 2: Knowledge COVID-19 ~ Knowledge General + Knowledge Influenza + Knowledge Cholera + age_factor +
region_factor + Gender
Res.Df RSS Df Sum of Sq F Pr(>F)
1 1497 41.170
2 1494 16.087 3 25.082 776.47 < 2.2e-16 ***

Significance. codes: 0 ‘***’ 0.001 ‘**’ 0.01 ‘*’ 0.05 ‘.’ 0.1 ‘ ’ 1

The absolute of the AIC for the COVID-19-19 Model knowledge was 2536 and differed substantially from the AIC for the basic model knowledge (=1127). Thus, the increase in model complexity was over-compensated by the increase in explained variance.

3.2 Assessing Knowledge of COVID-19 model assumptions

3.2.1 Assessment of multicollinearity:

	GVIF	Df	GVIF^(1/(2*Df))
Knowledge General	1.829131	1	1.352454
Knowledge Influenza	1.683658	1	1.297559
Knowledge Cholera	1.679109	1	1.295804
age_factor	1.091125	2	1.022042
region_factor	1.241433	5	1.021862
Gender	1.010686	1	1.005329

As all VIFs were well below 10, we did not assume multicollinearity. For the Knowledge variables in the IV set, Spearman intercorrelations ranged between 0.444 and 0.552 thus also not indicating biased estimators due to suppressor effects.

3.2.2 Independence of errors

```
lag Autocorrelation   D-W Statistic p-value
   1       0.1676298        1.663986          0
 Alternative hypothesis: rho! = 0
```

Durbin-Watson-Test indicated significant autocorrelations.

3.2.3 Normality of errors

Normality of errors was assessed for a random subsample (n=100) in order to avoid inflated significance values due to extremely low standard errors.The mean of the model residuals was 0.002, the median was 0.014 indicating a symmetric distribution. Neither skewness (-0.490) nor kurtosis (0.561) nor the Shapiro-Wilks test (0.974, p > 0.05) indicated a significant deviation from normality.

3.3 Per-case assessment of the knowledge model

3.3.1 Outliers

```
COVID-19Model_Knowledge$largeResiduals <- abs(rstandard(COVID-19Model_Knowledge)) > 2
sum(COVID-19Model_Knowledge$largeResiduals)/length(COVID-19Model_Knowledge$largeResiduals)
[1] 0.05046481
```

The rate of outliers with standardized residuals >2 was approx. 5 % thus indicating no elevated number of outliers.

3.3.2 Identifying Influential Cases

Influential single cases are classified as those with large residuals (see above) and simultaneously having an elevated leverage (above 3 times (k + 1) / n with k being the number of predictors and n being the number of observations) or simultaneously having an increased Cook's distance (>1; see Stevens, 2002).

```
COVID-19Model_Knowledge$large.cvr <-abs (COVID-19Model_Knowledge$cvr - 1) > 39/1506
sum(COVID-19Model_Knowledge$large.cvr)
[1] 69
sum(COVID-19Model_Knowledge$largeResiduals && COVID-19Model_Knowledge$large.cvr)
[1] 0
sum(COVID-19Model_Knowledge$largeResiduals && COVID-19Model_Knowledge$cooks.distance > 1)
[1] 0
```

As both criteria revealed no records exerting undue influence onto the results of the analysis, the result can well be understood as not biased by (groups of) observations with extreme data.

3.3 The extended knowledge of COVID-19 model

The knowledge of COVID-19 model was extended to assess the influences of the socio-demographic characteristics on the knowledge of COVID-19. Comparing the models for Knowledge on COVID-19

showed that all of the variables which were entered blockwise to the Knowledge-model differed significantly.

Analysis of Variance Table
Model 1: Knowledge COVID-19 ~ Knowledge General + Knowledge Influenza + Knowledge Cholera + age_factor +
region_factor + Gender
Model 2: Knowledge COVID-19 ~ Knowledge General + Knowledge Influenza + Knowledge Cholera + age_factor +
region_factor + Gender + Community
Model 3: Knowledge COVID-19 ~ Knowledge General + Knowledge Influenza + Knowledge Cholera + age_factor +
region_factor + Gender + Community + Education + Occ_factor
Model 4: Knowledge COVID-19 ~ Knowledge General + Knowledge Influenza + Knowledge Cholera + age_factor +
region_factor + Gender + Community + Education + Occ_factor +
Religion_factor

	Res.Df	RSS	Df	Sum of Sq	F	Pr(>F)
1	1483	16.012				
2	1482	15.542	1	0.46994	53.351	4.547e-13 ***
3	1479	13.317	3	2.22515	84.204	< 2.2e-16 ***
4	1476	13.001	3	0.31547	11.938	1.003e-07 ***

Significance. codes: 0 ‘***’ 0.001 ‘**’ 0.01 ‘*’ 0.05 ‘.’ 0.1 ‘ ’ 1

Beta coefficients and confidence intervals for different linear regression models for Knowledge about COVID-19 . **Bold indicates significance**.

Coefficient:	Basic Model	Community added	education and occupation added	religion added
General knowledge	**0.314**	**0.293**	**0.146**	**0.140**
Influenza knowledge	**0.262**	**0.254**	**0.111**	**0.103**
Cholera knowledge	**0.341**	**0.314**	**0.200**	**0.186**
Age (25-54)	**-0.051**	**-0.050**	**-0.051**	**-0.040**
Age (55 +)	**-0.055**	**-0.047**	**-0.024**	-0.009
Region (Ashanti)	**0.050**	**0.048**	**0.054**	**0.046**
Region (Western)	-0.006	-0.007	-0.023	-0.027
Region (Volta)	**0.052**	**0.053**	**0.048**	**0.044**
Region (Savannah)	**0.099**	**0.105**	**0.124**	**0.115**
Region (Upper East)	**0.080**	**0.076**	**0.079**	**0.075**
Gender	-0.014	-0.006	0.001	-0.004
Community		**-0.113**	**-0.124**	**-0.120**
Education			**0.394**	**0.396**

Formal occupation			-0.020	-0.025
Informal occupation			0.010	0.002
Christian				-0.008
Traditional				**-0.088**
None				-0.026

Knowledge: The explained variance increased from **64.3%** for the model presented above (important: with a limited number of records compared to the model presented above due to missingness for the higher order models) to **65.4%** by adding the community variable, to **70.3%** by further adding the variables education (significant at 0.001) and occupation (n.s. for both contrasts) and to **70.9%** by additionally adding the religion-Factor.

Comparing the abs of the AIC (2514, 2556, 2781 and 2811 – the same pattern was confirmed for the BIC's) revealed that solely the second step and in this step namely the education substantially contributed to the explained variance.

Section S4: Linear Multiple Regression Analysis: Influences on the practice against COVID-19

4.1 Assumption: Practices is the interesting dependent variable

lm (formula = P_COVID-19 ~ Knowledge COVID-19 + A_COVID-19 + +age_factor + region_factor + Gender, data = ID_regression)

Residuals:
Min 1Q Median 3Q Max
-0.36561 -0.08570 -0.00113 0.085200.42358

Coefficients	Estimate	Std.	Error	t- value	Pr(>|t|)	Boootstrapped 5% conf-limit
(Intercept)	0.1977532	0.0204876	9.652	<2e-16	***	0.1637
Knowledge of COVID-19	0.1467935	0.0193886	7.571	6.45e-14	***	0.1168
Age (25-54)	-0.0050847	0.0067574	-0.752	0.4519		n.s.
Age 55+	-0.0341089	0.0172180	-1.981	0.0478	*	-0.0049
Region (Ashanti)	0.0455975	0.0105711	4.313	1.71e-05	***	0.028
Region (Western)	-0.0137185	0.0112725	-1.217	0.2238		n.s.
Region (Volta)	-0.0013306	0.0110950	-0.120	0.9046		n.s.
Region (Savannah)	0.0051881	0.0112213	0.462	0.6439		n.s.
Region (Upper East)	0.0178485	0.0112858	1.582	0.1140		n.s.
Gender	0.0006018	0.0064451	0.093	0.9256		n.s.

Significance. codes: 0 ‘***’ 0.001 ‘**’ 0.01 ‘*’ 0.05 ‘.’ 0.1 ‘ ’ 1

Residual standard error: 0.1242 on 1491 degrees of freedom
(14 observations deleted due to missingness)
Multiple R-squared: 0.07546, Adjusted R-squared**: 0.06926**
F-statistic: 12.17 on 10 and 1491 DF, p-value: < 2.2e-16

The one-sided 5% Bootstrapped Confidence intervals led to the same statistical decisions as the t-Test did. Therefore, the alpha-error probabilities can well be assumed to be not heavily biased by the violation of the assumption of normality.

The model explained only **6.93%** of the variance of the practices from the knowledge on COVID-19 , the agefactor3 (comparing age group 55 and above to age group 15-24) and the regional factor 2 (comparing Ashanti to Greater Accra Region).

This Model was compared to a Basic Model, including only age, gender and region as independent variable. The basic model, although significant, explained only 3.47% (adjusted R^2) of the variance of the dependent variable. Although the model fit did not differ largely in terms of the explained variance, the models differed significantly.

Analysis of Variance Table

Model 1: P_COVID-19 ~ age_factor + region_factor + Gender
Model 2: P_COVID-19 ~ Knowledge COVID-19 + A_COVID-19 + age_factor + region_factor + Gender

	Res.Df	RSS	Df	Sum of Sq	F	Pr(>F)
1	1493	23.886				
2	1491	23.001	2	0.88506	28.687	5.96e-13

Significance. codes: 0 '***' 0.001 '**' 0.01 '*' 0.05 '.' 0.1 ' ' 1

Accordingly, the AIC for the COVID-19ModelPractices (absolute = 1990) differed only marginally from the AIC for the BasicModelPractices (=1938). Although significant, It remains questionable if the increase in model complexity is worth the marginal increase in explained variance.

4.2 Assessing practice model assumptions

4.2.1 Assessment of multicollinearity:

	GVIF	Df	GVIF^(1/(2*Df))
Knowledge of COVID-19	1.105230	1	1.051299
A_COVID-19	1.043969	1	1.021748
age_factor	1.094069	2	1.022730
region_factor	1.201545	5	1.018530
Gender	1.010665	1	1.005318

As all (G)VIFs were well below 10, we did not assume multicollinearity.

4.2.2 Independence of errors

lag	Autocorrelation	D-W Statistic	p-value
1	0.06146146	1.875658	0.014

Alternative hypothesis: rho! = 0

Durbin-Watson-Test indicated significant but only marginal autocorrelations.

4.2.3 Normality of errors

Normality of errors was assessed for a random subsample (n=100) in order to avoid inflated significance values due to extremely low standard errors.

The mean of the model residuals was -0.010, the median was -0.014 indicating a symmetric distribution. Neither skewness (0.256) nor kurtosis (-0.252) nor the Shapiro-Wilks test (0.989, $p > 0.05$) indicated a significant deviation from normality.

4.3 Per-case assessment of the knowledge model

4.3.1 Outliers:

```
> COVID-19Model_Practices$largeResiduals <- abs(rstandard (COVID-19ModelPractices)) > 2
> sum(COVID-19Model_Practices$largeResiduals)/length(COVID-19Model_Practices$largeResiduals)
[1] 0.04460719
```

The rate of outliers with standardized residuals >2 was approx. **4.5%** thus indicating no elevated number of outliers.

4.3.2 Identifying Influential cases

Influential single cases are classified as those with large residuals (see above) and simultaneously having an elevated leverage (above 3 times (k + 1) / n with k being the number of predictors and n being the number of observations) or simultaneously having an increased Cook's distance (>1; see Stevens, 2002).

```
> COVID-19Model_Practices$large.cvr <-abs(COVID-19Model_Practices$cvr - 1) > 33/1502
> sum(COVID-19Model_Practices$largeResiduals && COVID-19Model_Practices$large.cvr)
[1] 0
> sum(COVID-19Model_Practices$largeResiduals && COVID-19Model_Practices$cooks.distance > 1)
[1] 0
```

As both criteria revealed no records exerting undue influence onto the results of the analysis, the result can well be understood as not biased by (groups of) observations with extreme data.

Beta coefficients and confidence intervals for a linear regression of preventive practices onto knowledge about COVID-19

	Beta coefficient				
Coefficient:	95% CI lower limit	estimate	95% CI upper limit	Pr(>|t|)	Bootstrapped 5% confi-lmit
Knowledge of COVID-19	0.147	0.198	0.250	***	0.153
Age (25-54)	-0.071	-0.020	0.032		n.s.
Age (55 +)	-0.102	-0.051	-0.001	*	- 0.007
Region (Ashanti)	0.077	0.141	0.205	***	0.085
Region (Western)	-0.097	-0.037	0.023		n.s.
Region (Volta)	-0.065	-0.004	0.058		n.s.
Region (Savannah)	-0.048	0.015	0.078		n.s.
Region (Upper East)	-0.012	0.050	0.111		n.s.
Gender	-0.047	0.002	0.051		n.s.

4.4 The extended Practice against COVID-19 model

Model 1: P_COVID-19 ~ Knowledge COVID-19 + A_COVID-19 + age_factor + region_factor + Gender

Model 2: P_COVID-19 ~ Knowledge COVID-19 + A_COVID-19 + age_factor + region_factor + Gender + Community

Model 3: P_COVID-19 ~ Knowledge COVID-19 + A_COVID-19 + age_factor + region_factor + Gender + Community + Education + Occ_factor
Model 4: P_COVID-19 ~ Knowledge COVID-19 + A_COVID-19 + age_factor + region_factor + Gender + Community + Education + Occ_factor + Religion_factor

Res.Df	RSS	Df	Sum of Sq	F	Pr(>F)
1 1484	22.935				
2 1483	22.882	1	0.053218	3.4698	0.06270
3 1480	22.822	3	0.059983	1.3036	0.27168
4 1477	22.654	3	0.168331	3.6584	0.01206 *

Significance. codes: 0 ‘***’ 0.001 ‘**’ 0.01 ‘*’ 0.05 ‘.’ 0.1 ‘ ’ 1

For this model, only the last step significantly changed the level of explained variance. Nevertheless, despite significant, comparing the AIC’s of the models showed that the increase in explanatory power was nearly neutralized by the increased model complexity while for the BIC’s the more complex model would be classified as worse than the basic model.

	df	AIC
COVID-19Model_Practices	12	-1978.314
COVID-19Model_Practices_3	19	-1982.779
	df	BIC
COVID-19Model_Practices	12	-1914.596
COVID-19Model_Practices_3	19	-1881.892

Section S5: Questionnaire

5.1. Questionnaire Validation

This questionnaire was pretested and validated by experts. The validation group (n=84): 56.6% females, 42.2% males and 1.2% others 44.6%; rural and 55.4% urban; educational level 30.1% tertiary, 28.9% secondary, 14.5% basic, 10.8% school dropout and 15.7% no education; religion 69.9% Christianity, 20.5% Islamic, 6% Traditional and 3.6% others; occupation 42.2% informal sector, 26.5% formal sector and 31.3% no occupation; marital status 45.8% single, 41.0% married and 13.2% others. The face and content validities of the questionnaire were reviewed individually by three experts from the Department of Psychiatry, Komfo Anokye Teaching Hospital, Department of Pharmacy Practice, Faculty of Pharmacy and Pharmaceutical Sciences and Department of Pharmaceutical Chemistry, Faculty of Pharmacy & Pharmaceutical Sciences, Kwame Nkrumah University of Science & Technology, Kumasi, Ghana.

5.2. Questionnaire:

SOCIAL DEMOGRAPHICS:

Year of birth?……………….

Religion: [ ] Christianity [ ] Islamic [ ] Traditional [ ] None [ ] others, specify..

Education level: [ ] No education [ ] School dropout [ ] Basic education [ ] Secondary education
[ ] Tertiary education

Occupational class: [ ] Informal sector [ ] Formal sector [ ] None

Marital status: [ ] Single [ ] Married [ ] Divorced [ ] widowed [ ] prefer not to say

Gender: [ ] Male [ ] Female [ ] others [ ] prefer not to say

Region/Area:……… Community: …………… [ ] Urban [ ] Rural

Part 1: Knowledge:

General

1. What is an infectious disease? [ ] spontaneous
[ ] spread from infected bodies [ ] a spiritual disease [ ] life time disease [ ] no idea

2. Can you please give examples of infectious disease outbreak(s)? More than one option applies
[ ] spontaneous
[ ] Cholera [ ] Cancer [ ] COVID-19 [ ] Diabetes [ ] H1N1 Influenza /Flu
[ ] Ebola [ ] Hypertension [ ] Meningitis

H1N1 Influenza (Swine flu)

3. What kind of disease is H1N1 influenza (swine flu)? [ ] spontaneous
[ ] Respiratory disease [ ] Stomach disease [ ] Sexually transmitted disease [ ] No idea

4. H1N1 influenza (swine flu) Spread through……. [ ] spontaneous
[ ] Infected body droplets [ ] Genetic [ ] Sex [ ] Blood transfusion [ ] No idea

5. Signs and Symptoms of swine Flu is/are .. More than one option applies [ ] spontaneous
[ ] Fever [ ] Diarrhea [ ] Cough and sore throat [ ] Blood in urine [ ] No idea

6. How can swine Flu be prevented? More than one option applies [ ] spontaneous
[ ] Wearing face mask [ ] Spiritual protection [ ] maintaining personal hygiene
[ ] Social distancing [ ] Eating balance diet

Cholera

7. What kind of disease is Cholera? [ ] spontaneous
[ ] Respiratory disease [ ] Stomach disease [ ] Heart disease [ ] No idea

8. Cholera is spread through…… [ ] spontaneous
[ ] Genetic [ ] Ingestion of infected food/water [ ] Sex [ ] Blood transfusion [ ] no idea

9. Signs and symptoms of Cholera are………………… [ ] spontaneous
[ ] Fever [ ] Diarrhea & vomiting [ ] Cough & sore throat [ ] Blood in urine [ ] No idea

10. How can Cholera be prevented? More than one option applies [ ] spontaneous
[ ] Wearing face mask [ ] Environmental hygiene [ ] Maintaining personal hygiene
[ ] Spiritual protection [ ] Use safe water/eating well cooked food

COVID-19

11. In what way are these two existing diseases (cholera and flu) similar to COVID-19 in terms of prevention and limiting the spread? [ ] spontaneous
[ ] keeping personal hygiene [ ] wearing face mask [ ] eating well cooked food [ ] none

12. What kind of disease is COVID-19 ? More than one option applies [ ] spontaneous
[ ] a genetic disease [ ] a virus causing disease [ ] a novel disease [ ] chronic disease
[ ] linked with respiratory symptoms

13. What causes COVID-19 ? [ ] spontaneous

[ ] Curse/Evil spirits [ ] Pathogens/microorganisms [ ] No idea [ ] Physiological disorder

14. What is/are the classical signs and symptoms of COVID-19 ? More than one option applies [ ]
[ ] Headaches [ ] Fever [ ] Dizziness [ ] Coughing [ ] Diarrhea

15. COVID-19 spreads through….? More than one option applies [ ] spontaneous
[ ] Human contact [ ] Contaminated food [ ] Infected surfaces
[ ] Respiratory droplets [ ] Birth

16. Who is at risk of getting COVID-19 ? [ ] spontaneous
[ ] no one [ ] rich persons [ ] everyone [ ] non-Ghanaians [ ] no idea

17. What is/are the commonest practices to prevent spread of COVID-19 ? More than one option applies
[ ] wearing face masks [ ] hospital attendance [ ] spiritual practices [ ] social distancing

18. Do you know about COVID-19-17? [ ] Yes [ ] No [ ] spontaneous

19. Which of the following possible action(s) do you take or would you take when you suspect you have COVID-19 ? More than one option applies [ ] spontaneous
[ ] self-isolation [ ] Get tested [ ] Seek spiritual help [ ] Take medications [ ] Nothing

PART 3: PRACTICES:
20. Which of the following action(s) do you take personally to prevent yourself from getting COVID-19 ?

a. Wearing face mask	[ ]Yes	[ ] No
b. Regular hand washing	[ ] Yes	[ ] No
c. Seeking spiritual protection	[ ] Yes	[ ] No
d. Avoid social gatherings	[ ]Yes	[ ] No
e. Avoid traveling	[ ]Yes	[ ] No
f. Eating balance diet	[ ] Yes	[ ] No
g. Social distancing	[ ]Yes	[ ] No

21. Have you used any medication (prophylaxis) to prevent being infected with COVID-19 ?
[ ] Yes [ ] No

22. Have you taken vaccination against COVID-19 ? [ ] Yes [ ] No

23. Do you seek updated information about COVID-19 ? [ ] Yes [ ] No

24. Have you tested for COVID-19 ? [ ] Yes [ ] No

25. Do you always wear face marks when going out to public places? [ ] Yes [ ] No

26. Would you take vaccination against COVID-19 if one is developed and approved for use?
[ ] Yes [ ] No

Section S6: Test for Representativeness

6.1 By Region

```
smp_reg <- as.integer(ftable(ID_raw$`Region/Area`))

pop_reg <- c(5055900, 5924500, 2214700, 1907700, 594700, 1302700)

smp_reg <- as.data.frame(cbind(smp_reg))

region_names <- c("Accra", "Ashanti", "Western", "Volta", "Savanna", "Upper east" )

rownames(smp_reg) <- region_names

colnames(smp_reg) <- c("sample")

reg_chisq <- chisq.test(smp_reg, p = pop_reg, rescale.p = TRUE)

reg_chisq


Chi-squared test for given probabilities


data:  smp_reg

X-squared = 1000.4, df = 5, p-value < 2.2e-16
```

Highly significant differences between sample and population, Therefore, further analysis:

```
# calculate for the contribution of vells in % to the Chi-Square:

# residuals^2/chi-square

# from: http://www.sthda.com/english/wiki/chi-square-test-of-independence-in-r

smp_reg$contrib <- round(100* reg_chisq$residuals^2/reg_chisq$statistic,3)


corrplot(as.matrix(smp_reg$contrib), is.corr = FALSE, addCoef.col = NULL,

    type = "full", tl.pos = "lt", cl.pos = "n")
```

Highest contribution to the deviation for region 5 = Savannah

6.2 By Gender

Important: This is somewhat in “Off-limits” test because you compare the sample from the regions which were included to the overall Ghanaian population. The result is only meaningful if there are no other structural/socio-demographic/economic/religious/educational/etc. Therefore, we calculated this test twice, first for the overall population and below for the population in the regions.

Relative to the overall population in Ghana

```
smp_gen <- as.integer(ftable(ID_raw$Gender))
# limit comparison to "male" and "female"
smp_gen <- smp_gen[1:2]
pop_gen <- c(8364700, 8635450)

smp_gen <- as.data.frame(cbind(smp_gen))
gender_names <- c("Male", "Female")
rownames(smp_gen) <- gender_names
colnames(smp_gen) <- c("sample")

gen_chisq <- chisq.test(smp_gen, p = pop_gen, rescale.p = TRUE)
gen_chisq

        Chi-squared test for given probabilities

data:  smp_gen
X-squared = 2.3077, df = 1, p-value = 0.1287
```

Non-significant deviation by gender, Therefore, stopping here

By Gender in relation to overall population in Ghana

```
smp_gen <- as.integer(ftable(ID_raw$Gender))
# limit comparison to "male" and "female"
smp_gen <- smp_gen[1:2]
pop_gen <- c(8364700, 8635450)

smp_gen <- as.data.frame(cbind(smp_gen))
gender_names <- c("Male", "Female")
rownames(smp_gen) <- gender_names
```

```
colnames(smp_gen) <- c("sample")

gen_chisq <- chisq.test(smp_gen, p = pop_gen, rescale.p = TRUE)
gen_chisq

        Chi-squared test for given probabilities

data:  smp_gen
X-squared = 2.3077, df = 1, p-value = 0.1287
```

Non-significant deviation by gender for the overall population, therefore, stopping here

Relative to the overall population in the studied regions

```
pop_gen <- c(5208284, 6477156)   # population in the studied regions
gen_chisq <- chisq.test(smp_gen, p = pop_gen, rescale.p = TRUE)
gen_chisq
        Chi-squared test for given probabilities
data:  smp_gen
X-squared = 26.609, df = 1, p-value = 2.491e-07
```

Highly significant difference.

	Sample		Population		
	Raw figure		percentage	Raw figure	percentage
Male	776	51.1	5,208,284	44.6	
Female	741	48.8	6,477,156	55.4	

Males are massively overrepresented in relation to the regional population.

6.3 By age groups, relative to the studied regions

```
smp_age <- as.integer(ftable(ID_raw$YoB))
pop_age <- c(3471487, 5892536, 695.558, 1625859)   # population in the studied regions
smp_age <- as.data.frame(cbind(smp_age))
age_names <- c("15-24", "25 - 54", "55 - 64", "65 - ")
```

```
rownames(smp_age) <- age_names
colnames(smp_age) <- c("sample")

age_chisq <- chisq.test(smp_age, p = pop_age, rescale.p = TRUE)
age_chisq
```

```
        Chi-squared test for given probabilities
data:  smp_age
X-squared = 14077, df = 3, p-value < 2.2e-16
```

Highly significant difference, therefore performing further analysis as to the contribution to the difference

	Sample		Population				
	Raw figure		Percentage	Raw figure		Percentage	Contrib to the deviation [%]
15-24	699	44.8	3471487	29.7	36.7		
25 - 54	800	51.3	5892536	50.4	0.1		
55 - 64	37	2.4	695558	6.0	10.3		
65 -	23	1.5	1625859	13.9	53.0		

Under-representation mostly of the oldest (65 -) and the youngest (15-24) age groups. With these one-dimensional differences between sample and population distribution, We decided not to calculate for a multi-dimensional test as this would for sure also end up with significant but according to the level of detail (Region * Gender * Age group) meaningless results. As a result:

For descriptive analyses, weights were calculated to correct the estimators to match the population distribution by region, gender and age group in the studied regions. This was nevertheless problematic:

In region 5, "Upper east", there were no respondents in the age classes “55 – 64” and “65 –“ for both genders and in region 2, “Western”, there were no female respondents aged “65 –“ (see red formatted figures in the table below)

Additional problem may arise from very low frequencies in some of the cells, e.g. for males and females in Area 0, “Accra” in age group “65-“ (see tables below)

a. Sample distribution by region, gender and age

	Male				Female			
Regions	15 - 24	25 - 54	55 - 64	65 -	15 - 24	25 - 54	55 - 64	65 -

Greater Accra	45	115	5	1	33	84	8	2
Ashanti	90	57	3	3	90	49	4	2
Western	38	46	3	5	46	71	6	0
Volta	56	66	1	2	53	56	1	2
Savanna	56	71	4	4	44	60	1	2
Upper East	59	45	0	0	67	60	0	0

As a result, to calculate the weights for the cells in this three-dimensional distribution, weights from the one-dimensional marginal distributions by region, age, and gender were separately calculated and cross-multiplied into the three-dimensional weight table.

Normed weights to mean = 1, Weights to correct for differences between sample and population distribution by region, gender and age

	Male				Female			
Regions	15 - 24	25 - 54	55 - 64	65 -	15 - 24	25 - 54	55 - 64	65 -
Greater Accra	0.283	0.417	1.065	3.897	0.368	0.542	1.386	5.069
Ashanti	0.326	0.480	1.227	4.490	0.424	0.624	1.596	5.841
Western	0.169	0.249	0.636	2.327	0.220	0.323	0.827	3.026
Volta	0.132	0.194	0.497	1.818	0.172	0.253	0.646	2.365
Savanna	0.040	0.059	0.152	0.555	0.052	0.077	0.197	0.722
Upper East	0.092	0.136	0.348	1.274	0.120	0.177	0.453	1.657

With these weights, 46 records were not included in the descriptive analysis as their gender was coded 3 = “prefer not to say”. One person additionally had not indicated his/her age group.

For the inferential analyses, region, gender and age group were always included as IV’s in order to reduce error variance and not to have the results confounded by the differing distributions.

Basically, a decision is needed at that point if the descriptive should only be calculated for the studied regions or for the whole Ghanaian population. The second case would be an “off limits” estimation and it validity not only depends on the representativeness according to the known parameters

but also to other, unknown parameters like differences between studied and non-studied regions in terms of e.g. education, religion, economy, (see above).
